# Exploring the role of the immune microenvironment in hepatocellular carcinoma: Implications for immunotherapy and drug resistance

**DOI:** 10.7554/eLife.95009

**Published:** 2024-08-15

**Authors:** Yumin Fu, Xinyu Guo, Linmao Sun, Tianming Cui, Chenghui Wu, Jiabei Wang, Yao Liu, Lianxin Liu

**Affiliations:** 1 Department of Hepatobiliary Surgery, Centre for Leading Medicine and Advanced Technologies of IHM, The First Affiliated Hospital of USTC, Division of Life Sciences and Medicine, University of Science and Technology of China Hefei China; 2 Anhui Provincial Key Laboratory of Hepatopancreatobiliary Surgery Hefei China; 3 Anhui Provincial Clinical Research Center for Hepatobiliary Diseases Hefei China; 4 Department of General Surgery, Key Laboratory of Hepatosplenic Surgery, Ministry of Education, The First Affiliated Hospital of Harbin Medical University Harbin China; Dalian University of Technology China; Shengjing Hospital of China Medical University China

**Keywords:** HCC, tumor immune microenvironment, immunoresistance, immunotherapy

## Abstract

Hepatocellular carcinoma (HCC), the most common type of liver tumor, is a leading cause of cancer-related deaths, and the incidence of liver cancer is still increasing worldwide. Curative hepatectomy or liver transplantation is only indicated for a small population of patients with early-stage HCC. However, most patients with HCC are not candidates for radical resection due to disease progression, leading to the choice of the conventional tyrosine kinase inhibitor drug sorafenib as first-line treatment. In the past few years, immunotherapy, mainly immune checkpoint inhibitors (ICIs), has revolutionized the clinical strategy for HCC. Combination therapy with ICIs has proven more effective than sorafenib, and clinical trials have been conducted to apply these therapies to patients. Despite significant progress in immunotherapy, the molecular mechanisms behind it remain unclear, and immune resistance is often challenging to overcome. Several studies have pointed out that the complex intercellular communication network in the immune microenvironment of HCC regulates tumor escape and drug resistance to immune response. This underscores the urgent need to analyze the immune microenvironment of HCC. This review describes the immunosuppressive cell populations in the immune microenvironment of HCC, as well as the related clinical trials, aiming to provide insights for the next generation of precision immunotherapy.

## Introduction

Primary liver cancer is currently the fourth most common malignant tumor and the second leading cause of cancer-related deaths in China, posing a significant threat to the lives and health of the population (Sung et al., 2021; Llovet et al., 2021b; Villanueva, 2019). Treatment options, such as hepatectomy or liver transplantation, are only suitable for patients in the early stages, with approximately 50–60% of liver cancer patients eventually requiring systemic therapy. For over a decade, kinase inhibitor therapy has been the main treatment for advanced liver cancer, including first-line drugs such as sorafenib and lenvatinib (in China, doxorubicin), with a median overall survival of 11–14 months. However, this therapy still cannot effectively control progressive liver cancer and prevent its recurrence. With the advancements and clinical application of immunology research, immuno-oncology has revolutionized tumor treatment. For instance, by combining anti-PD-L1/PD-1 and cytotoxic T lymphocyte antigen 4 (CTLA-4) monoclonal antibodies (mAbs) with anti-vascular endothelial growth factor (VEGF) bevacizumab, the median survival of liver cancer patients can reach 19 months, bringing new hope to the clinical treatment of liver cancer (Llovet et al., 2021a; Llovet et al., 2018; Finn et al., 2020; Qin et al., 2021). Nevertheless, the lack of accurate prediction and systematic research and response to primary and secondary resistance mechanisms in key populations has led to poor outcomes (El-Khoueiry et al., 2017; Zhu et al., 2018a; Yau et al., 2019). It is generally assumed that a positive response to immunotherapy usually depends on the dynamic interaction between tumor cells and the tumor microenvironment (TME). Increasing studies have shown that the inhibitory changes and heterogeneous characteristics of TME have a great impact on tumor development, differential efficacy, and drug resistance. In this review, we will analyze the cells, mechanisms of action, and related clinical trials in the immune microenvironment of hepatocellular carcinoma (HCC) that regulate immune resistance, providing new insights for future HCC treatment.

### Liver Cancer-Immune Microenvironment

The liver is a key frontline immune tissue that maintains systemic homeostasis by having relatively high immune tolerance to foreign antigens, especially those of intestinal origin. A large population of key cells with major immunosuppressive effects are involved in immune evasion in HCC, including regulatory T (Treg) cells, myeloid-derived suppressor cells (MDSCs), tumor-associated macrophages (TAMs), tumor-associated neutrophils (TANs), and dendritic cells (DCs) (Llovet et al., 2022a; Llovet et al., 2022b).

#### Immunosuppressive Lymphocytes

**Figure 1.**
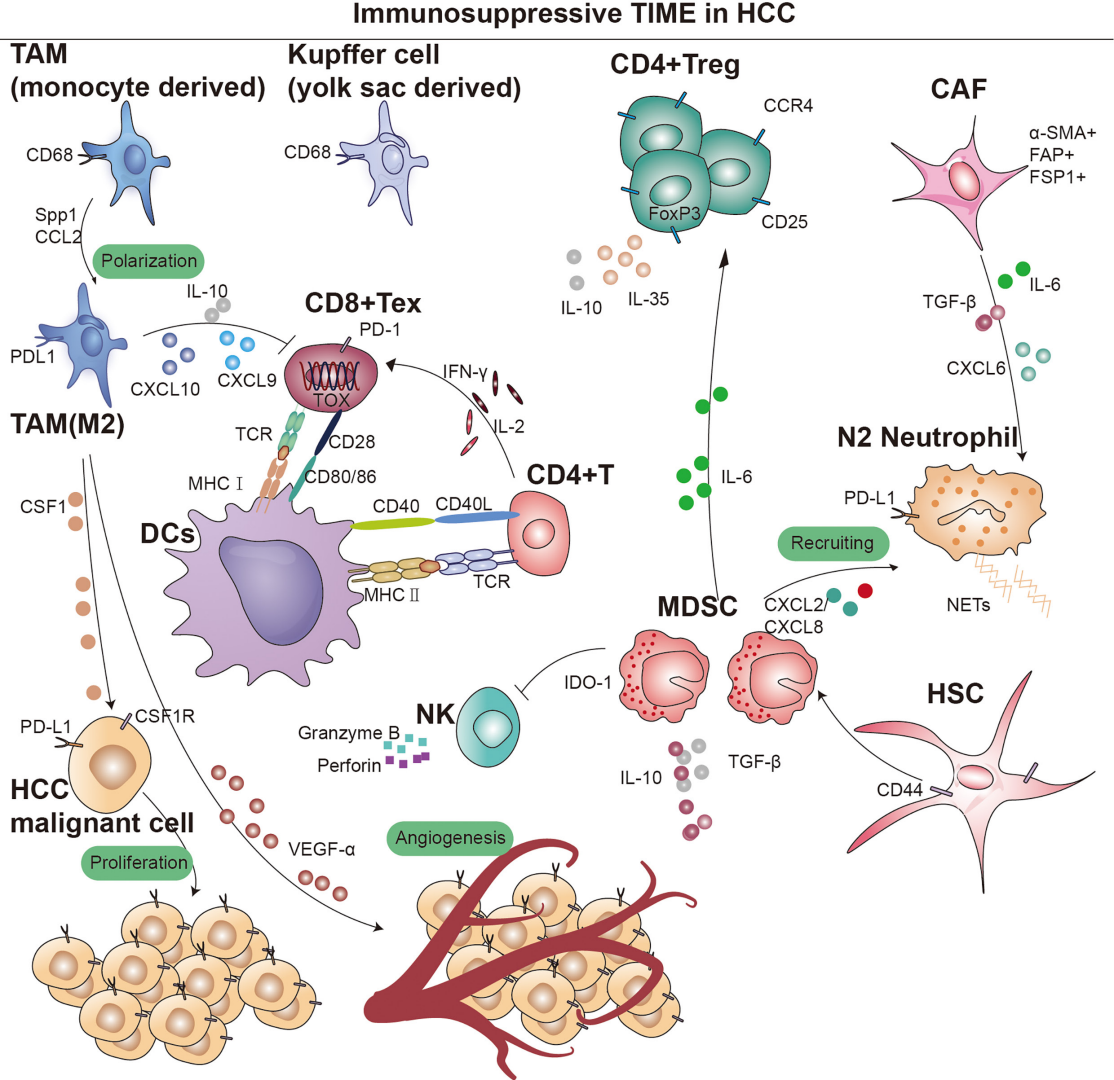
Overview of immunosupressive cells in the hepatocellular carcinoma (HCC) immune microenvironment. Key cell types and cellular component implicated in immune surveillance are indicated in this figure. TAM, tumor-associated macrophage; MDSC, myeloid-derived suppressor cells; CD4^+^ Treg, regulatory CD4^+^ T cells; CD8^+^ Tex, exhausted CD8^+^ T cells; CAF, cancer-associated fibroblast; DCs, dendritic cells; TCR, T-cell receptor; IDO, indoleamine 2,3-dioxygenase; MHC I, major histocompatibility complex class I; MHC II, major histocompatibility complex class II.

During acute inflammation and injury, naive CD8^+^ T cells can differentiate into effector CD8^+^ T cells, TEFF, to response after receiving antigen stimulation. And part of TEFF will form long-lived self-renewable memory CD8^+^ T cells, TMEM, which can produce rapid immune response during infection. However, during tumor immunity, T cells differentiate into exhausted CD8^+^ T cells, TEX, with gradual loss of cytokines, high expression of inhibitory markers (PD-1, Tim-3, LAG3, TIGIT, and 2B4), metabolic alterations, and decreased proliferative potential and viability in response to persistent antigenic stimulation (Sen et al., 2016). The sustained high expression of inhibitory receptors represents a core characteristic of CD8^+^ T-cell exhaustion. The surface inhibitory receptors on T cells, immune regulatory cell populations such as Treg, immunoregulatory antigen-presenting cells (APCs), MDSCs, and soluble molecules such as immunosuppressive cytokines IL-10 and transforming growth factor (TGF)-β, inflammatory cytokine I type IFN (IFN-I), γ chain cytokines IL-2 and IL-7, all play a role in regulating the depletion of CD8^+^ T cells (McLane et al., 2019; Cheng et al., 2021; Kurachi, 2019). Typically, the more inhibitory receptors that are co-expressed by exhausted CD8^+^ T cells, the more severe the exhaustion (Blackburn et al., 2009). TEX minimizes immune-mediated pathological damage by limiting tumor immunity, and this limiting function can be thought of as sustained progression or worsening of the disease. Given that an effective immune response is almost entirely contingent upon functional T cells, the depletion of CD8^+^ T cells within TME represents a primary cause of immunotherapy resistance in HCC. Mary Philip and Andrea Schietinger found that thymocyte selection-associated HMG box protein, TOX, is highly expressed in tumor-specific CD8^+^ T cells (Figure 1). Overexpression of TOX in vitro induced a depletion phenotype in CD8^+^ T cells (Scott et al., 2019). Although the process of T-cell exhaustion can hinder anti-tumor immunity, this dysfunctional state can be reversed by targeting programmed cell death protein 1 (PD-1) and CTLA-4, thereby enhancing tumor immunity. Consequently, reversing human T-cell exhaustion is a crucial mechanism through which patients receiving PD-1 and CTLA-4 pathway drugs achieve significant anti-tumor effects (Blackburn et al., 2009; Schietinger and Greenberg, 2014; Said et al., 2010).

Regulatory T cells (Tregs) are a subset of lymphocytes known for their potent immunosuppressive functions, characterized by the constitutive expression of CD25 and CTLA-4 on their cell surface, as well as the presence of the transcription factor Forkhead box P3 (Foxp3) in their nucleus. However, their accumulation in tumors suppresses anti-tumor immunity (Tay et al., 2023). Subpopulations of Treg can modulate the expression of inhibitory receptors and depletion signaling pathways of CD8^+^ T cells through IL-10 and IL-35, which leads to diminished tumor killing capacity (Figure 1; Sawant et al., 2019). Sawant et al. deleted IL-10 or IL-35 in Tregs resulted in attenuated tumor growth and a concomitant dramatic reduction of TEX in the tumor (Ribas and Wolchok, 2018; Pauken and Wherry, 2015). Evidence from certain studies hints at a possible linkage between the PD-1 pathway and the generation of IL-10. This linkage is thought to be facilitated by the induction of IL-10 synthesis in monocytes as a result of the engagement between PD-1 and PD-L1 (Said et al., 2010). Further studies revealed that immunosuppressive cytokines such as IL-10 and IL-35, dependent on the BLIMP-1 pathway, promote CD8^+^ T-cell exhaustion (Turnis et al., 2016). The functional plasticity observed in Tregs is considered a crucial aspect of their cellular lineage. Sawant et al. have identified distinct subpopulations of Tregs within tumors that secrete IL-10 or IL-35, yet these phenotypes are transient, indicating that Tregs can transition between these states (Panduro et al., 2016). Despite the elucidation of the interconversion potential of IL-10 and IL-35 secretion by Tregs within tumors, the signaling mechanisms governing this conversion remain poorly understood. Investigating the conditions that facilitate this conversion would offer insights into regulating the therapeutic targeting of this cell population (Chan et al., 2016; Mumm et al., 2011). Sequencing analysis and single-cell mapping of tumor and paracancerous tissues showed a significant decrease in CTL/Treg ratio in HCC tissues, indicating that effector cells in the immune microenvironment of HCC tumor tissues had shifted from an activated to a suppressed state (Ally et al., 2017). In addition, TGF-β is associated with the promotion of Treg production, differentiation, and the resulting inhibition of CD8^+^ T cells. Thus, inhibition of TGF-β with the specific inhibitor SM-16 reduces Treg infiltration, leading to HCC tumor regression (Figure 1; Shen et al., 2015). Research indicates that CCR4^+^ Tregs acquire immunosuppressive stem-like properties through long-term chromatin reprogramming. CCR4 antagonist can impede intratumoral Tregs aggregation, thereby overcoming resistance (Gao et al., 2022). A similar mechanism has previously been reported for the Treg cell-specific marker CCR8 (Whiteside et al., 2021; Roose et al., 2021). Additionally, single-cell sequencing analysis of specimens from 29 HCC patients undergoing immunotherapy indicates a high infiltration of Tregs alongside PD-1^+^CD8^+^ T cells (Magen et al., 2023). Furthermore, studies have shown that CD4^+^CD25^+^ Tregs obtained from patients, when co-cultured with CD8^+^ T cells, can suppress their proliferation, activation, as well as the production of granzyme B and perforin (Fu et al., 2007).

#### Myeloid-Derived Suppressor Cells

MDSCs are immature bone marrow-derived cells and are therefore highly heterogeneous, with monomorphonuclear and polymorphonuclear distinctions, and can be categorized into two major subgroups: granulocytic (G-) MDSCs or polymorphonuclear (PMN)-like MDSCs and monocytic (M-) MDSCs. In mice, PMN-MDSCs are characterized as CD11b^+^Ly6G^+^Ly6C^low^ cells, while M-MDSCs are identified as CD11b^+^Ly6G^-^Ly6C^high^ cells. Considering the absence of Gr-1 in humans, PMN-MDSCs are defined as CD11b^+^CD14^-^CD15^+^ or CD11b^+^CD14^-^CD66b^+^ cells, whereas M-MDSCs are designated as CD11b^+^CD14^+^HLA-DR^-/low^CD15^-^ cells (Bronte et al., 2016). The recruitment of MDSCs to the TME is crucial for establishing an immunosuppressive niche. Specifically, PMN-MDSCs are primarily recruited through CXC chemokines, including CXCL1, CXCL2, and CXCL5 (Groth et al., 2021; Lim et al., 2020). On the other hand, M-MDSCs migrate toward the TME via the CCL2-CCR2 axis (Lim et al., 2020). Notably, CCR5 ligands (CCL3, CCL4, and CCL5) play a vital role in the recruitment of both MDSC subsets (Blattner et al., 2018). MDSCs are present in small numbers in the peripheral blood of healthy individuals, yet their numbers increase dramatically after tumorigenesis and migrate through the peripheral blood circulation to the site of the lesion. MDSCs have been reported to significantly upregulate tumor-associated macrophages, TAM receptor tyrosine kinases (RTKs) (TYRO3, AXL, MERTK, etc.), and corresponding ligands, leading to immunosuppression (Holtzhausen et al., 2019). MDSCs can bind to the CD40 receptor and express high levels of indoleamine 2,3-dioxygenase (IDO) or arginase 1 (ARG1), inducing a significant production of IDO1, leading to the expansion of Tregs (Figure 1; Yu et al., 2013; Zoso et al., 2014). MDSCs lack the necessary neutral amino acid transporter, which results in a significant limitation of the essential nutrients required for T-cell activation. Furthermore, MDSCs also deprive T cells of the cysteine necessary for activation, thereby compromise anti-tumor function (Srivastava et al., 2010). Additionally, iNOS is upregulated in the cytoplasm of MDSCs, where large amounts of L-arginine are metabolized to nitric oxide (NO) and L-citrulline, and NO drives negative communication between T cells, including interfering with IL-2R signaling and catalyzing the T-cell receptor (TCR), ultimately leading to the suppression of T-cell function (Lu et al., 2019; Lee-Chang et al., 2019; Bodogai et al., 2015). In addition, hypoxia-inducible factor 1α (HIF-1α) upregulation enhances the differentiation of M-MDSCs into immunosuppressive TAMs (van Vlerken-Ysla et al., 2023; Kwak et al., 2020). M-MDSCs also secrete IL-10, which suppresses DCs and promotes Tregs development. MDSCs induce immunocompetence by interacting with PD-1 receptors on T cells. Furthermore, other immune checkpoint molecules like VISTA, Gal-9, and CD155 have been implicated in MDSC-mediated immunosuppression (Wu et al., 2019b; Johnston et al., 2014; Zhang et al., 2020; Limagne et al., 2019; Le Mercier et al., 2014; Wang et al., 2018; Noman et al., 2014; Antonios et al., 2017). Preclinical evidence also suggests that PMN-MDSCs are susceptible to ferroptosis, releasing oxidized lipids that impair T-cell function. Moreover, extracellular vesicles (EVs) from PMN-MDSCs inhibit NK cell-mediated anti-tumor activity (Tumino et al., 2021; Burke et al., 2014).

Extensive research has focused on how MDSCs contribute to the immunosuppressive microenvironment. Primarily, PMN-MDSCs demonstrate upregulated expression of ARG1 and reactive oxygen species (ROS), associated with their immunosuppressive functions, while M-MDSCs are characterized by the expression of immunoregulatory molecules like TGF-β, IL-10, and PD-L1 (Gabrilovich, 2017; Gabrilovich et al., 2012; Veglia et al., 2021). However, MDSCs display significant heterogeneity across subsets and among patients, leading to variability in the expression of these molecules. Therefore, understanding MDSCs’ heterogeneity and their immunosuppressive mechanisms remains a critical challenge in HCC immunotherapy.

#### Tumor-Associated Macrophages

Macrophages are versatile immune cells, involved in tissue homeostasis, pathogen defense, and wound healing (Wynn et al., 2013). Those present in tumor tissues or the microenvironment, known as TAMs, exhibit distinct subsets based on differential gene expression. TAMs can be categorized into TAM1 (FOLR2^+^), TAM2 (SPP1^+^), and TAM3 (MT1G^+^), with TAM1 further divided into CD163^high^/CD206^high^ and TAM2/TAM3 into CD163^low^/CD206^low^ subsets (Sharma et al., 2020). Various markers distinguish these subsets, such as SPP1 and FOLR2 (Sharma et al., 2020; Finn et al., 2020; Sharma et al., 2012; Pliner et al., 2019). Krasniewski et al., 2022 utilized single-cell sequencing and flow cytometry, identifying four subsets of macrophages by membrane markers LYVE1 and MHCII, indicating their dynamic polarization. Analysis of TCGA data showed a correlation between TAMs and poor patient prognosis (Zhang et al., 2019a). This may be due to the fact that TAMs inhibit T cells from recognizing and killing tumor cells (Condamine et al., 2016). We later found that upregulation of macrophage SPP1 expression in the hypoxic TME interacts with CAFs to stimulate extracellular matrix (ECM) reprogramming, which together form a tumor immune barrier (TIB) structure in TME (Sharma et al., 2020). Blockade of SPP1 or macrophage-specific knockdown of SPP1 in HCC model breaks the immunotherapeutic barrier TIB and sensitizes tumor cells to immunotherapy (Liu et al., 2023). Chemokines like CSF1 and CCL2 in the TME recruit peripheral blood monocytes, where they ultimately differentiate into immunosuppressive M2 macrophages (Figure 1; Kang et al., 2011; Eggert et al., 2016; Li et al., 2017). TME with TAM-like features (expression of APOE, C1QA, C1QB, and TREM2, and high expression of SLC40A1 and GPNMB) (Lavin et al., 2017) was significantly associated with poor patient prognostic status, with SLC40A1 encoding transferrin. This observation aligns with recent findings by Mertens et al., 2018 regarding the involvement of iron metabolism in macrophage phenotypic differentiation. In a preclinical model of liver tumor metastasis, activated antigen-specific Fas^+^CD8^+^ T cells undergo apoptosis after interacting with FasL^+^CD11b^+^F4/80^+^ macrophages, culminating in the formation of an ‘immune desert’ (Yu et al., 2021). It was also found that fetal liver-associated PLVAP ECs, CD163 macrophages, and TIGIT Tregs were specifically enriched in tumor tissues (Zhang et al., 2019a; Rantakari et al., 2016), while CD8^+^ T cells and NKT cells were predominantly present in paraneoplastic tissues. Furthermore, TAMs not only dampen T-cell activity but also facilitate tumor progression and metastasis through signaling pathways like JAK2/STAT3/miR-506-3p/FoxQ1 (Wei et al., 2019).

In addition to NOTCH and VEGF signaling pathways, Petty et al. identified the Hedgehog signaling pathway’s involvement in TAM polarization toward M2. Tumor cells secrete sonic hedgehog, driving M2 polarization, and produce CXCL9 and CXCL10, inhibiting CD8 T-cell recruitment into the TME (Figure 1). Furthermore, TAMs can secrete VEGF-α to facilitate stromal angiogenesis, thereby further promoting the progression of HCC (Banerjee et al., 2023).

The M2 macrophage-targeting peptide (M2pep), designed to selectively target M2 macrophages, has emerged as a promising agent for inducing tumor cell and M2 macrophage toxicity. Studies have demonstrated that M2pep can exert its effect without impacting M1 macrophages (Kakoschky et al., 2018; Ngambenjawong et al., 2016; Cieslewicz et al., 2013). FDA-approved zoledronic acid, a third-generation amino-bisphosphonate agent, has demonstrated its ability to reverse the polarity of TAMs from M2-like to M1-like by attenuating the production of IL-10, VEGF, and MMP9 while restoring iNOS expression (Comito et al., 2017; Chen et al., 2021). An additional agent repolarizing TAMs to the M1 phenotype is CP-870,893, a CD40 agonist. Activation of macrophages via CD40, characteristic of the M1 phenotype, leads to increased release of pro-inflammatory cytokines and upregulated expression of antigen-presenting molecules such as MHC-II (Baer et al., 2016). TAM RTKs are also implicated in resistance to immune checkpoint inhibitors (ICIs). Notably, AXL has been identified as one of the top potential drug targets to overcome resistance to ICIs in bioinformatic analyses of large omics datasets from clinical studies and CRISPR screens (Li et al., 2022; Jiang et al., 2021). Bemcentinib, the first of these agents, demonstrates favorable safety and efficacy profiles in phase I and II studies, other selective AXL TKIs, such as DS-1205c and SLC-391, are also being evaluated in clinical trials (DeRyckere et al., 2023; Valle et al., 2021). Administration of CD40 mAb has been demonstrated to induce macrophage-dependent tumor regression in mice. The tolerability and efficacy of CP-870,893, alone or in combination with chemotherapy, have been assessed in multiple clinical trials (Beatty et al., 2011). Inhibitors targeting the CCL2/CCR2 or CSF-1/CSF-1R signaling axis have shown promise in reducing macrophage accumulation at tumor sites. Emactuzumab (RG7155), a novel humanized antibody targeting CSF-1R, has been observed to decrease the number of TAMs expressing CSF-1R in tumor lesions (Ries et al., 2014). Additionally, the oral tyrosine kinase inhibitor of CSF-1R, pexidartinib (PLX3397), has demonstrated encouraging results in early clinical trials (Tap et al., 2019). Other pharmaceutical agents, including the CCL2 inhibitor bindarit, anti-CCL2 mAb carlumab, CSF1 inhibitor GW2580, and dequalinium-14, CD40 antagonist CP-870,893, have also shown anti-tumor effects by reducing macrophage infiltration (DeNardo and Ruffell, 2019). Furthermore, the density of TAMs often correlates with the density of vessels in tumor tissues. Consequently, TAMs exert a significant influence on the efficacy of anti-angiogenic therapy. Notably, VEGF antagonists induce vascular normalization, which concurrently remodels the TAM phenotype (DeNardo and Ruffell, 2019; Mantovani et al., 2017). Despite promising preclinical data, the translational benefits of TAM-targeting agents in clinical studies have been somewhat modest. Further investigations are warranted to evaluate their therapeutic efficacy as monotherapy or in combination therapy settings.

#### Tumor-Associated Neutrophils

Neutrophils are innate immune cells that are the first cells to infiltrate tissues in infections, injuries, or tumors. Several studies have demonstrated that the level of TAN infiltration is positively correlated with poor prognosis in patients (Coffelt et al., 2016). TANs come in two different flavors: anti-tumorigenic (N1) or protumorigenic (N2). Protumorigenic N2 TANs have the capacity to form decondensed chromatin studded with granular and some cytoplasmic proteins, called neutrophil extracellular traps (NETs), known to support tumor growth (Cheng et al., 2018; Arvanitakis et al., 2021).

In addition, several studies have demonstrated interactions between tumor cells, TANs, and cancer-associated fibroblasts (CAFs) in HCC progression. CAF can inhibit neutrophil function via the SDF-1αglycolytic activation/CXCR4/IL-6 pathway, and also secrete cardiotrophin-like cytokine factor 1 (CLCF1), which mediates the tumor’s expression of CXCL6 and TGF-β, respectively, which are responsible for neutrophil recruitment and polarization toward N2 (Figure 1).

N2 neutrophils induce a stem cell phenotype in HCC cells, and co-culture induces the secretion of CXCL5, which further promotes tumorigenesis (Zhou et al., 2019; Zhou et al., 2012). Several studies have shown that a dysregulated neutrophil-to-lymphocyte ratio is strongly associated with the prognosis of patients with HCC. This may be due to the release of TGF-β by neutrophils, which affect immune regulation and tumor angiogenesis (Gonzalez-Sanchez et al., 2021). Galunisertib/LY2157299, a novel TGF‐β receptor 1 kinase inhibitor, is being investigated in phase II trials in combination with nivolumab (NCT02423343), sorafenib, or ramucirumab (NCT02240433, NCT02178358, and NCT01246986) (Donne and Lujambio, 2023).

#### Dendritic Cells

DCs are important APCs in the immune system and have migratory properties that allow them to present antigens to immune killer cells in tissues and lymph nodes (Heras-Murillo et al., 2024). For example, the activation of CD8^+^ T cells depends on the early activation of DCs by CD4^+^ T (Th) helper cell (Figure 1). The process of antigen presentation requires the formation of an immune synapse (Jenne and Kubes, 2013). The complete immune synapse has three regulatory steps: DCs are required to present antigens on MHC molecules to T cells individually and interact with co-stimulatory molecules of the TNF superfamily (CD40L/CD40, 4-1BBL/4-1BB, CD27/CD70, CD30L/CD30, and HVEM/LIGHT) to trigger stimulation of CD8^+^ T cell activation (Figure 1; Thaiss et al., 2011). And one of the main mechanisms of cancer cell immune escape is to disrupt this immune synapse by expressing inhibitory ligands for T-cell activation, e.g., PD-1, CTLA-4; lymphocyte activation 3, LAG3; hepatitis A virus cell receptor 2, TIM3 (McLane et al., 2019).

DC populations can be divided into several categories based on their developmental spectrum and stage of differentiation: conventional DCs (cDCs), plasmacytoid DCs (pDCs; CD303^+^CD304^+^, secreting type I IFN), and inflammatory DCs (Jellinger, 2022).

Several studies have observed reduced circulating pDCs and cDCs in the peripheral blood of HCC patients compared to healthy controls, and lower expression of co-stimulatory molecules on these DCs (Pirillo et al., 2023). BDCA2^+^ pDCs in tumor tissue were associated with high alpha-fetoprotein (AFP) levels, advanced tumor-node-metastasis staging, and increased tumor infiltration by Tregs and IL-17-producing cells (Zhou et al., 2017). In vitro experiments revealed that pDCs induced the differentiation of CD4^+^ T cells into IL-10-producing Tr1 cells. In addition, tumor cDCs from patients with HCC were found to express inhibitory ligands such as PD-L1, Gal9 (ligand for TIM3), MHC-II (LAG3), CD86 and CD80 (CTLA-4) (Sun et al., 2021). Therapeutic strategies targeting DCs, such as overt immunotherapy and DC vaccines, are available to restore anti-tumor responses. A meta-analysis highlighted that immunotherapy based on DCs cells could improve CD4^+^ T/CD8^+^ T ratio while ensuring safety (Chen et al., 2018). streptococcal-derived DC-OK432 that produce large amounts of Th1 cytokines (IL-12 and IFN-γ) and enhance cytotoxic T-cell activity via CD40/CD40L co-stimulatory molecules (Nakamoto et al., 2011), which is effective in combination immunotherapy (Teng et al., 2021). Mouse liver cancer model treated with DC vaccine in combination with PD-1 inhibitor has longer OS and significantly reduced tumor volume (Nakai and Matsumura, 2021). There is also clinical trial data that suggests this combination therapy could be a potential treatment for cancer patients (Vogt et al., 2021).

#### Cancer-Associated Fibroblasts

Within stromal components, CAFs play a pivotal role, upregulating multiple membrane surface molecular markers including alpha-smooth muscle actin, fibroblast activation protein, fibroblast-specific protein 1, platelet-derived growth factor receptor (PDGFR)-α/β, and vimentin (Figure 1; Giraldo et al., 2019; Barrett and Puré, 2020). While a small subset of CAFs may restrict tumor growth, the majority of CAF populations have been consistently shown across numerous studies to promote tumor cell proliferation (Mizutani et al., 2019; Fiori et al., 2019; Hinshaw and Shevde, 2019; Joshi et al., 2021).

(1) Tumorigenesis leads to ECM reprogramming by matrix metalloproteinases (MMPs), fostering the creation of a specific extracellular milieu conducive to cancer progression (Chen and Song, 2019). CAFs are central to maintaining ECM homeostasis, synthesizing and secreting ECM proteins under pathological conditions (Miles and Sikes, 2014).

CAFs rely on protease- and force-mediated ECM remodeling to facilitate tumor cell invasion and metastasis. Moreover, the hypoxic TME regulates ECM remodeling by CAFs, with HIF-1α-expressing CAFs promoting tumorigenesis and metastasis (Calvo et al., 2013; Gilkes et al., 2014).

(2) The most thoroughly studied paracrine pathway of CAFs is TGF-β (Mariathasan et al., 2018). TGF-β binds to transmembrane surface receptor serine/threonine kinase complexes, inducing downstream Smad complex activation, which regulates target gene expression (Colak and Ten Dijke, 2017; Mu et al., 2012). Recently, a strong association has emerged between stem cell-like properties and the mesenchymal-like phenotype of tumor cells (Morel et al., 2012; Todaro et al., 2014). Cancer cells undergoing epithelial-mesenchymal transition (EMT) often acquire stem cell-like traits, facilitated by upregulation of TGF-β and secretion of SPP1, allowing simultaneous acquisition of stemness and completion of EMT (Fessler et al., 2016; Heldin et al., 2012). Additionally, various pathways such as MAPK, PI3K/Akt, Wnt/β-catenin, hepatocyte growth factor (HGF)/c-MET, and JAK/STAT contribute to tumor cell phenotypic transitions, forming intricate feedback loops crucial for tumor survival, stemness, EMT, metastasis, and clonal potential (Ding et al., 2018; Comoglio et al., 2018; Lozano et al., 2023). Patients with heightened expression of signaling molecules like TGF-β, MAPK, and PI3K/Akt typically exhibit disease progression and poor prognosis (Calon et al., 2015).

(3) CAFs also exert a significant influence on tumor angiogenesis, primarily through the upregulation of pro-angiogenic factors such as VEGF and PDGF (Fukumura et al., 1998). In addition, CAFs secrete high levels of CXCL12 to recruit endothelial progenitors to participate in revascularization (Feig et al., 2013). Furthermore, CAFs contribute to tumor progression by modulating the expression of various proteases involved in ECM remodeling, thereby promoting tumor cell colonization and metastasis (Bronisz et al., 2012). NF-κB plays an important role in CAFs, mediating the upregulation of proteases such as COX2, CXCL1, CXCL2, CYR61/CCN1, IL-1β, IL-6, and osteopontin, thus promote tumor growth, recruit macrophages, and form tumor vasculature (Erez et al., 2010).

Multiple studies have highlighted the importance of cross-talk between tumor cells, TANs, and CAFs in influencing HCC progression. CAFs can inhibit neutrophil function via the SDF-1α/CXCR4/IL-6 pathway, which in turn induces the expression of CD66b, PD-L1, CXCL8/IL-8, TNF, and CCL2, and can inhibit T-cell function and proliferation in vitro (Johnson et al., 2018). Secretion of CLCF1 by CAFs can mediate tumoral expression of CXCL6 and TGF-β, responsible for neutrophil recruitment and N2 type polarization, respectively (Colak and Ten Dijke, 2017). These multifaceted interactions underscore the critical role of CAFs in tumor progression and suggest their potential as therapeutic targets.

### Interactions Between Immunosuppressive Cell Populations and Tumor Cells

Cancer cells manipulate their microenvironment by modulating immune cells and the ECM, creating an environment conducive to tumor sustenance. This intercellular communication occurs through direct cell-to-cell interactions involving adhesion molecules like integrins, cadherins, and selectins, as well as through paracrine signaling pathways (Dobie and Skropeta, 2021). Notably, the PD-L1/PD-1 pathway is exploited by tumor cells to evade immune surveillance (van Niel et al., 2022; Lucotti et al., 2022). Additionally, tumor cells remodel their microenvironment by releasing cytokines, chemokines, growth factors, and proteases. The ECM serves as a critical medium through receptors such as integrins and CD44 for intercellular communication, providing a surface for cell adhesion and migration and sequestering secreted molecules (Dey et al., 2021; Xiao et al., 2019). Immune cells, particularly M2 polarized macrophages, play a pivotal role in shaping the tumor stroma by secreting growth factors like fibroblast growth factor, and PDGF-β, and by producing TGF-β, which triggers fibroblast activation and collagen deposition (Dhanasekaran et al., 2016; Llovet et al., 2016). The impact of these factors is amplified by the ability of various leukocytes to secrete matrix proteolytic enzymes such as MMP9, which liberate ECM-bound factors capable of inducing stromal mitogenesis (Munitz and Levi‐Schaffer, 2004). As a consequence, macrophages emerge as critical orchestrators within the TME, capable of inducing myofibroblast differentiation, fostering fibrosis, and sustaining stromal cell viability.

The interaction between myeloid and stromal cells has received considerable attention, but other immune cell infiltrates, such as Tregs, TH2, TH17, and NKT cells, also significantly influence TME (Coulouarn and Clément, 2014). Understanding these interactions is crucial for elucidating the immunosuppressive mechanisms disrupting the cancer immunity cycle and improving responsiveness to immunotherapy.

### The Relationship Between TME Components and Immunotherapy Resistance

#### Lymphocytes and Immunotherapy Resistance

As previously discussed, the depletion of CD8^+^ T cells in TME stands as a primary impediment to effective tumor immunotherapy, given its heavy reliance on T-cell functionality. Upregulation of immune checkpoints is a leading mechanism behind immunotherapy resistance, directly linked to the exhaustion of CD8^+^ T cells. Ma et al.’s research underscores that disease progression in HCC correlates with an enrichment of PD-1^high^ CD8^+^ T cells within the TME (Ma et al., 2019; Wang et al., 2023). Targeting immune checkpoints such as PD-L1, TIM-3, or LAG-3 with ICIs in HCC can reverse the exhausted state of infiltrating T cells within the TME, thereby enabling them to exert their anti-tumor functions. Pro-inflammatory cytokines, particularly IFN-I, within the TME initially stimulate T-cell activation, thus fostering an environment conducive to tumor immunotherapy (Yu et al., 2022). However, recent findings reveal that sustained signaling of inflammatory cytokines, specifically IFN-I, exacerbates the terminal exhaustion of CD8^+^ T cells, leading to resistance to ICIs therapy and a poorer prognosis in cancer patients (Zheng et al., 2021). Chronic stimulation by the inflammatory cytokine IFN-I can induce lipid peroxidation, intensify the exhaustion program of CD8^+^ T cells, and dampen the effector function of exhausted CD8^+^ T cells. Notably, studies have demonstrated that treatment with either anti-PD-1 or anti-type I interferon receptor-1 (IFNAR-1) alone failed to improve the survival rate in a mouse model of HCC. However, combined therapy with anti-PD-1 and anti-IFNAR-1 significantly prolonged the survival rate of the mice (Chen et al., 2022a).

#### MDSCs and Immunotherapy Resistance

MDSCs are capable of producing MMP9. Kumar et al., 2016 observed in a tumor model of immunodeficient mice, co-injected with fibroblasts and tumor cells, an increased expression of MMP9 and angiogenic factors. MDSCs and TAMs were attracted to the tumor site through the specific chemotactic action of CCL2, enabling the tumor to evade immune surveillance. Crucially, the caspase recruitment domain protein 9 (CARD9) regulates tumor growth in the TME by modulating IDO in collaboration with MDSCs (Wu et al., 2019a). MDSCs secrete cytokines like IL-6, IL-10, and IL-23, promoting cell survival, as evidenced in studies co-culturing MDSCs with multiple myeloma cells (De Veirman et al., 2019). Additionally, MDSCs facilitate tumor progression by activating Tregs, inhibiting tumor immunity via immunosuppressive cytokines (IL-10, TGF-β), and producing NO and ROS (Lin et al., 2019). Notably, MDSC-derived EVs contribute to immunosuppression through increased PD-L1 expression and TGF-β secretion (Lin et al., 2019; Fleming et al., 2019; Iwata et al., 2016). Moreover, high ARG1 levels in TME deplete L-arginine, impairing T-cell function, while MDSC-derived iNOS and NOX2 induce oxidative stress, inhibiting T-cell proliferation and migration (Feng et al., 2018; Fiaschi and Chiarugi, 2012). Besides, NO and peroxynitrite derived from MDSCs can inhibit T-cell migration to tumor sites by reducing the expression of adhesion molecules such as E-selectin on endothelial cells and disrupting chemokine-mediated recruitment (Gehad et al., 2012). Additionally, MDSCs can inhibit the expression of IFN-γ and TNF by NK cells, as well as antibody-dependent cellular cytotoxicity, through the generation of NO (Stiff et al., 2018). Ultimately, MDSCs directly suppress effector T cells and natural killer cells, while also fostering an immunosuppressive milieu by stimulating other suppressive immune cell types.

#### TAMs and Immunotherapy Resistance

TAMs exert their immunosuppressive function by expressing immune inhibitory receptors such as PD-L1/2 and CD80/CD86, which lead to the depletion of T cells. Interaction between PD-L1 on TAMs and PD-1 on T cells inhibits downstream signaling transduction of TCR, consequently inducing T-cell exhaustion (Chen et al., 2012; Liu et al., 2020). Additionally, TAMs can reshape TME by secreting immunosuppressive factors such as VISTA, TGF-β, IDO, and anti-inflammatory mediators (Hmeljak et al., 2018; Llosa et al., 2015). Furthermore, TAMs can induce an immunosuppressive niche involving Tregs, CAFs, DCs, MDSCs, and other cells. Our previous research has demonstrated that TAMs upregulate the expression of SPP1 in hypoxic microenvironments and interact with CAFs, leading to metabolic reprogramming and the formation of a ‘TIB’ within the microenvironment. This distinctive spatial structure weakens the therapeutic efficacy of immune-based therapies (Hashimoto et al., 2016). Furthermore, multiple studies indicate that TAMs can directly impact tumor cells by enhancing their resistance to apoptosis-inducing drugs. Angiopoietin-2 serves as a regulator of vascular integrity and is functionally linked to TAMs, endowing tumor cells with an anti-VEGF escape capability (Coffelt et al., 2010; Mantovani et al., 2017). Additionally, studies have reported that the sustained glycolytic activation in TAMs microenvironment may deprive immune effector cells of glucose, thereby attenuating the efficacy of immunotherapy (Chang et al., 2015). Arlauckas et al., 2017 found that TAM populations expressing high levels of Fcγ receptors can sequester immune checkpoint blockers, preventing their pharmacological interaction with their targets, thus contributing to the development of resistance. Concurrently, Neubert et al., 2018 observed that PD-1 therapy-induced activation of T cells results in increased secretion of CSF1, promoting M2 polarization of TAMs, which emerges as a crucial determinant of resistance. Consequently, this provides compelling rationale for the combination inhibition of CSF1R and PD-1 (Zhu et al., 2014). As previously discussed, the metabolic programming of TAMs and their cross-talk with various immune components within the TME represent promising therapeutic targets for the development of novel immunotherapies.

#### TANs and Immunotherapy Resistance

Increasing evidence suggests that TANs contribute to immunoresistance in HCC. Neutrophils have been shown to release various cytokines, thereby promoting inflammatory responses and immune modulation within the TME. These cytokines include HGF, oncostatin M, β2-integrin, neutrophil elastase, MMP9, and VEGF (Kuang et al., 2011). Furthermore, multiple studies have indicated that TANs can directly promote cancer cell growth, migration, and invasion. Additionally, Fan et al. found that TANs can also secrete chemokines such as CCL2 and CCL17, thereby modulating the microenvironment and recruiting macrophages to infiltrate tumor sites, thus advancing HCC progression (Zhou et al., 2016). Murine model studies have shown that the depletion of TANs significantly augments the therapeutic efficacy of sorafenib (De Bock et al., 2011). Research utilizing samples from patients with HCC undergoing anti-PD-1 immunotherapy and multi-site specimens from animal models has enabled the construction of a single-cell neutrophil atlas. This atlas revealed a higher prevalence of CD10^+^ALPL^+^ neutrophils in anti-PD-1-resistant patients. These neutrophils exhibit an immunosuppressive phenotype by promoting irreversible T-cell exhaustion and reducing their cytotoxicity. Furthermore, these neutrophils originate from tumor cell reprogramming (Meng et al., 2023).

Upon specific stimuli, neutrophils can extrude NETs, a web-like structure comprising DNA, histones, and antimicrobial proteins, closely linked to cancer progression (Yang et al., 2020). In the context of liver fibrosis, increased NETs in the tumor-associated ECM, together with collagen type I (Col1) enveloping tumor cells, hinder their physical contact with cytotoxic T cells, thereby impairing ICI response. Additionally, shield-like structures formed by NETs and Col1 around HCC cells trap and directly deplete activated T cells through proteases and immunosuppressive molecules decorated on the chromatin scaffold of NETs (Schauer et al., 2014; Kaltenmeier et al., 2021). In addition to protecting HCC cells from invasion by adherent activated T cells, we have also observed that the synergistic action of liver fibrosis-associated ECM, Col1 and NETs prevents distant T-cell infiltration into the tumor area, thereby creating a region locally enriched in neutrophils/NETs but depleted of T cells. Other studies have documented an antagonistic distribution of neutrophils/NETs and T cells in the liver fibrosis-associated ECM/Col1-enriched area (Rømer et al., 2021; Chen et al., 2022b). The enrichment of NETs and the scarcity of T cells in the TME also elucidate the more aggressive nature of intrahepatic metastasis in patients with liver cirrhosis-associated HCC.

#### DCs and Immunotherapy Resistance

DCs play pivotal roles in eliciting and modulating immune responses. However, under steady-state conditions devoid of pro-inflammatory stimuli, the activation of T cells by DCs results in T-cell tolerance (Jones et al., 2016). The activation of CD8^+^ T cells relies on the immunological synapse, where a complete immunological synapse is formed by DCs presenting antigens on MHC molecules and engaging in interactions with co-stimulatory molecules, thereby triggering the production of cytokines crucial for the proliferation and differentiation of CD8^+^ T cells. Dysfunction in DC-mediated antigen presentation and subsequent T-cell suppression constitute the core mechanisms underlying immune evasion in HCC (Barry et al., 2018).

#### CAFs and Immunotherapy Resistance

Öhlund and colleagues have delineated two distinct subsets of CAFs, namely inflammatory CAFs and myofibroblastic CAFs (myCAFs) (Northey et al., 2017). Utilizing single-cell RNA sequencing technology, Kieffer et al. further identified two subgroups within myCAFs, ECM-myCAF and TGF-β-myCAF, which play critical roles in shaping the immunosuppressive milieu and conferring resistance to immunotherapy (Costa et al., 2018). Their study demonstrated that ECM-myCAFs stimulate the expression of PD-1 and CTLA-4 proteins on the surface of CD4^+^CD25^+^ T lymphocytes, while PD-1^+^ CTLA-4^+^ Tregs can reciprocally alter the proportion of TGF-β-myCAFs by converting ECM-myCAFs into TGF-β-myCAFs (Costa et al., 2018). Concurrently, CAFs produce abundant collagen, potentially erecting physical barriers that impede the migration of adaptive immune cells to new antigen-presenting sites (Mariathasan et al., 2018). Additionally, studies have suggested that the activation of pathways associated with smooth muscle cell contraction and innate immune cell activation within myCAFs may be linked to the establishment of an immunosuppressive microenvironment, potentially explaining their resistance (Galbo et al., 2021).

### Current Status of Immunotherapy for HCC

**Figure 2.**
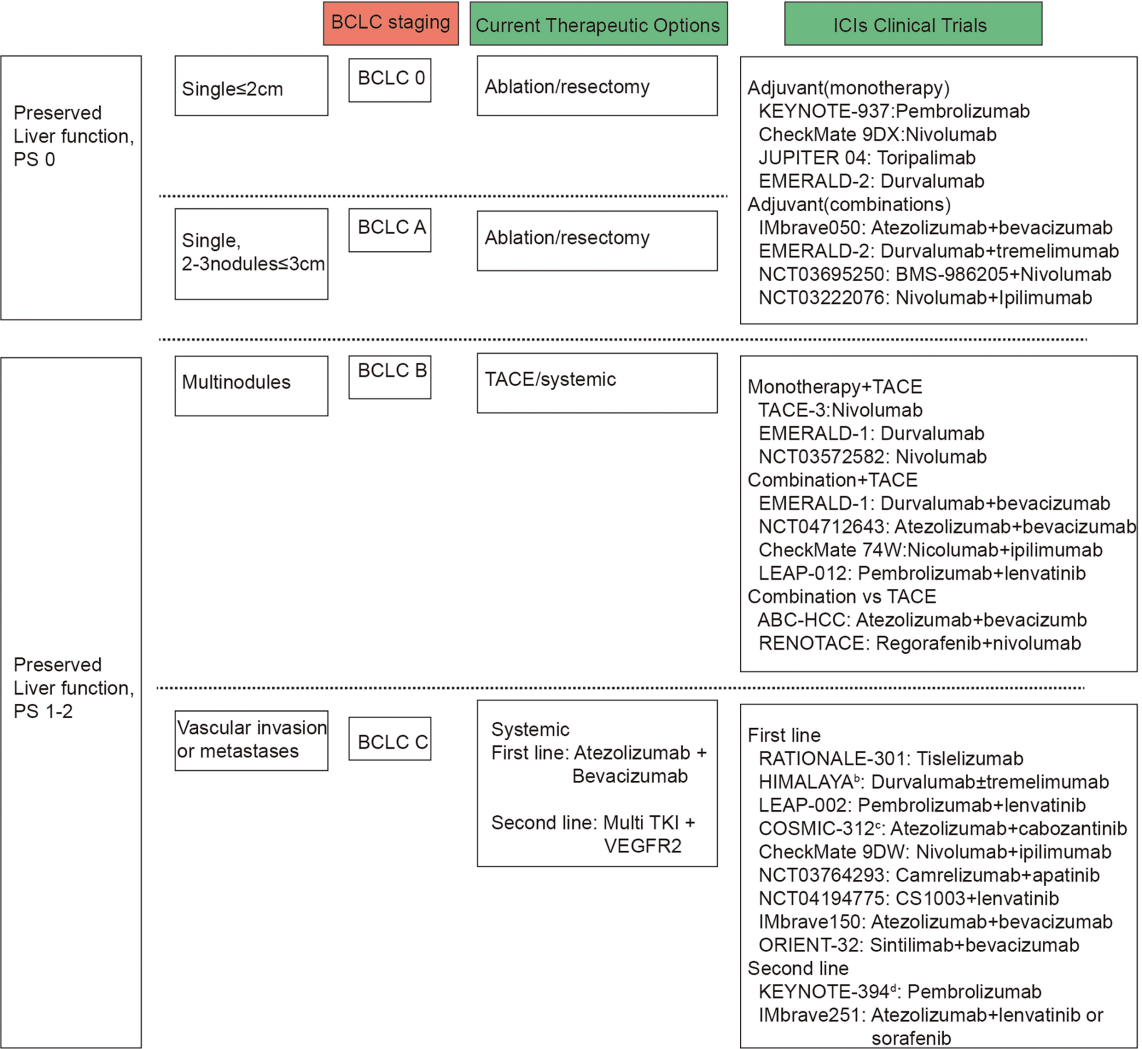
Barcelona Clinic Liver Cancer (BCLC) and immune checkpoint inhibitors (ICIs) clinical trials by BCLC staging. BCLC staging are based on tumor number and size, vascular invasion or metastases, preserved liver function and performance status. Presented on the right side of the figure are the clinical trials carried out for patients with liver cancer, categorized according to the BCLC staging system, alongside the primary therapeutic interventions applied.

The latest Barcelona Clinic Liver Cancer (BCLC) staging system is established based on tumor burden, liver function, performance status, and cancer-related symptoms. This staging system enables clinicians to tailor treatment strategies and prognostic predictions, ultimately improving patient management and outcomes (Figure 2). Surgical resection, radiofrequency ablation, and transarterial chemoembolization (TACE) are available for early and intermediate stage patients, while systemic therapy, including immunotherapy, is indicated for BCLC C stage patients (Figure 2).

Clinical trials exploring adjuvant and neoadjuvant immunotherapies in early- or intermediate-stage HCC have shown promising results. Phase III studies are evaluating adjuvant anti-PD-1/PD-L1 antibody monotherapy or co-inhibition of the PD-L1-PD-1 and VEGF-VEGFR pathways after surgery. Pilot neoadjuvant studies assessing anti-PD-1 antibodies alone or with anti-CTLA-4 antibodies are also underway (Pinato et al., 2021; Topalian et al., 2020; Haber et al., 2021; Lee et al., 2015; Liu et al., 2016). Neoadjuvant immunotherapies, including nivolumab, ipilimumab, cemiplimab, and cabozantinib, have demonstrated encouraging outcomes in early-stage HCC, with increased T-cell infiltration and pathological complete response rates observed (Topalian et al., 2019; Galle et al., 2018). However, these treatments have not been integrated into clinical guidelines due to trial design limitations and lack of validation studies. Further validation through phase III trials is needed before incorporating these strategies into clinical practice. Despite multiple clinical trials incorporating early-stage BCLC patients into immunotherapy cohorts, BCLC stage C patients consistently represent the majority population (78–91%) (Bruix et al., 2021).

In intermediate-stage HCC, the synergy between immunotherapy and locoregional therapies is better understood compared to surgery. Immunotherapy following locoregional treatments like tumor ablation, TACE, or transarterial radioembolization shows promise due to increased antigen presentation resulting from tumor cell destruction (Gudd et al., 2021; Dolladille et al., 2020). Subsequent immune effects, known as ‘abscopal’ effects, can be augmented with ICIs. Tremelimumab immunotherapy has shown increased infiltration of tumor CD8^+^ T cells in HCC patients, suggesting its combination with subtotal ablation or TACE (Pinato et al., 2020; Nogueira et al., 2019). Preliminary results from the PETAL study on pembrolizumab post-TACE indicate tolerability. Combinations of other ICIs with TACE are in phase III trials (Kelley et al., 2021; Fukumura et al., 2018). Adding anti-angiogenic drugs to locoregional therapy and ICIs may enhance efficacy, with ongoing phase III trials investigating this approach. Comparative studies between systemic therapies like atezolizumab and bevacizumab versus TACE in intermediate-stage HCC are underway in phase III trials like RENOTACE and ABC-HCC.

Immunotherapy is also applicable to patients who have failed initial treatment but remain in stages preceding BCLC stage C. Since the initiation of the Imbrave150 study, immunotherapy has been formally incorporated into the first-line treatment of advanced liver cancer patients (Figure 2). The latest advancements and strategies in immunotherapy are as follows.

#### ICIs Combination Therapy

PD-1/PD-L1 inhibitors combined with CTLA-4 inhibitors, such as nivolumab in combination with ipilimumab, and tremelimumab plus durvalumab regimen. Additionally, phase II studies investigating the combination of immunosuppressive agents with TACE or 90Y radioembolization are also underway.

#### Immunotherapy Combined with Targeted Agents

Currently, several combination regimens have been approved, including atezolizumab plus bevacizumab, pembrolizumab plus lenvatinib, camrelizumab plus apatinib, and sintilimab plus bevacizumab analogues. Additionally, ongoing clinical studies include the TALENTACE trial evaluating the combination of atezolizumab with sorafenib or lenvatinib in phase III clinical research for patients who have received prior treatment with atezolizumab plus bevacizumab, the SHR-1210-III-310 trial assessing the combination of camrelizumab with apatinib as first-line therapy for advanced HCC, and the LEAP-012 trial investigating the combination of pembrolizumab with regorafenib for patients with advanced HCC who have experienced immunotherapy failure or progression (Llovet et al., 2022b; Kudo et al., 2022).

#### Combining Immunotherapy with Local Therapy

Several preclinical studies suggest that incorporating radiotherapy into immunosuppressive therapy not only directly kills tumor cells but also enhances immune surveillance by activating immune responses, thereby improving immune resistance (Bu et al., 2023; Wang et al., 2022). Research has reported that PD-L1 combined with radiotherapy can restore the normal function of CD8 T cells in tumor tissue, thereby exerting anti-tumor immune effects. In a study combining nivolumab with radiotherapy, all patients responded to the combination therapy. Interestingly, the development and application of some nanoparticles can induce cancer cell death, further activating the immune system (Zhang et al., 2024).

#### Oncolytic Virus Therapy

Oncolytic virus (OV) therapy involves the selection or genetic engineering of viruses to preferentially infect, replicate within, and lyse tumor cells. The principle behind this therapy is that the overexpression of receptors on the surface of tumor cells and various signaling pathways associated with viral clearance enable OV to enter tumor cells and induce their destruction (Shi et al., 2020). Nakatake et al., 2018 investigated the anti-tumor activity and immune response of the third-generation HSV T-01 in animal models. Additionally, the recombinant OVs M1-c6v1 and VG161 have received FDA approval, and there are several ongoing clinical studies evaluating their combination with immunotherapy.

#### Tumor Vaccines

These vaccines work by amplifying tumor-specific T-cell responses through active immunization. They primarily target AFP and glypican 3 (GPC3) in HCC (Hong et al., 2014). UCPVax (a telomerase-derived CD4^+^ helper T-cell-inducing tumor vaccine) in combination with atezolizumab plus bevacizumab has entered phase II clinical trials (Vienot et al., 2023).

#### Cell-Based Therapies

Cell-based therapies refer to the process of genetically modifying patient immune cells to express chimeric antigen receptors (CARs) and then reinfusing them back into the patient’s body. These engineered cells are capable of binding to specific tumor antigens, stimulating immune-mediated destruction of tumor cells, thereby enhancing overall tumor-specific anti-tumor effects (Akce et al., 2018; Zhang et al., 2019b). Major cell-based therapies include NK cell therapy, TIL therapy, and CAR T-cell therapy, among others. In HCC, adoptive cell therapies primarily target GPC3, with multiple related studies progressing into phase I clinical trials (Rochigneux et al., 2021; Zhu et al., 2018b; Zheng et al., 2022).

Frequently employed immunotherapies encompass combination therapy with ICIs and ICIs plus targeted agents. This section highlights those that have transitioned into practical clinical application, focusing on ICI therapy and combination approaches (Table 1).

**Table 1.** Clinical trials of target inhibitors or immune checkpoints in the tumor microenvironment.

	Clinical trial	Phase	Target	Intervention/treatment	State	Output	PMID
First-line ICI therapy	IMBrave150(NCT03434379)	III	PD-L1;VEGF	Atezolizumab: 1200 mg IV d1, Q3W Bevacizumab: 15 mg/kg IV d1, Q3W	Completion (Nov 17, 2022)	ORR:27.3% OS:19.2m PFS:6.83m	34902530 34051880 32402160
	ORIENT-32(NCT03794440)	II/III	PD-1;VEGF	Sintilimab: 200 mg IV d1, Q3W IBI305: 15 mg/kg IV d1, Q3W	Completion (Jan 22, 2021)	Not yet posted	34143971
	CheckMate 459(NCT02576509)	I/II	PD-1	Nivolumab: Specified dose on specified days	Active	OS:16.39m vs 14.69m ORR:15.4% vs 7.0% PFS:3.68m vs 3.75m	34914889
	GO30140(NCT02715531)	I	PD-L1;VEGF	5-FU: 400 mg/m^2^ IV, followed by 2400 mg/m^2^ IV, Q2W Atezolizumab: 1200 mg Q3W Bevacizumab: 15 mg/kg Q3W	Completion (May 31, 2021)	OS:17.1m PFS:7.3m	32502443
	KEYNOTE-524(NCT03713593)	III	VEGFR2 (KDR)/VEGFR3;PD-1	Lenvatinib: PO, QD Pembrolizumab: 200 mg, Q3W	Active	PFS:8.2 vs 8.1 OS:21.2 vs 19.0 ORR:26.1 vs 17.5	
	RATIONALE 208(NCT03419897)	III	PD-1	Tislelizumab: 200 mg IV d1, Q3W	Completion (July 6, 2022)	ORR：13.3% OS:13.2m	36872927 34518988
Second-line ICI therapy	KEYNOTE-224(NCT02702401)	III	PD-1	Pembrolizumab: 200 mg IV, Q3W	Completion (Sept 22, 2021)	ORR:18.3% vs 4.4% OS:13.9m vs 10.6m ORR:18.3% vs 4.4%	31790344
	KEYNOTE-240(NCT02702401)	III	PD-1	Pembrolizumab	Completion (Sept 22, 2021)	PFS:3.0m vs 2.8m OS:13.9m vs 10.6m ORR:18.3% vs 4.4%	31790344
	CheckMate 040(NCT01658878)	I/II	PD-1	Nivolumab: IV, on specific days	Active	Not yet posted	34051329 33001135 32710922 31176752 28434648
	RESCUE(NCT03463876)	II	PD-1;VEGF	SHR-1210: 200 mg IV, Q2W apatinib: 250 mg PO,QD	Completion (Mar 10, 2021)	ORR:34.3% PFS:5.7m	33087333
	NCT02519348	II	PD-L1;CTLA-4;VEGF	Tremelimumab 300 mg plus durvalumab 1500 mg, followed by durvalumab 1500 mg Q4W, durvalumab monotherapy 1500 mg Q4W, tremelimumab monotherapy 750 mg Q4W (7doses) and then tremelimumab Q7W, or tremelimumab 75 mg once every 4 weeks plus durvalumab 1500 mg once every 4 weeks (4 doses), followed by durvalumab 1500 mg Q4W	Active	ORR:17.05% PFS:3.52m	34292792
	NCT01008358	II	CTLA-4	Tremelimumab: 15 mg/kg on day 1 of every 90-day cycle	Completion (May 2012)	OS:8.2m	23466307
	NCT03695250	I/II	IDO1 PD-1	IDO1 Inhibitor BMS-986205: PO QD on days 1–14 Nivolumab: IV over 30 min on day 1	Completion (March 12, 2021)	ORR:12.5%	
	NCT02989922	II	PD-1	Camrelizumab (3 mg/kg,Q3W)	Completion (June 2019)	OS:13·8m	32112738 35101942

#### First-Line Setting

The results of the IMbrave150 demonstrated that atezolizumab in combination with bevacizumab achieved significant improvements in both overall and progression-free survival, with median OS not yet reached in the combination therapy group and median OS of 13.2 months (10.4 months~NE) in the sorafenib group (Cheng et al., 2022). Analysis of 194 Chinese patients from a subgroup revealed a hazard ratio (HR) for OS of 0.44, suggesting a 56% relative reduction in the risk of death with the immunotherapy combination regimen versus sorafenib, and a 6-month survival rate of 86.6% (Table 1; Finn et al., 2020; Galle et al., 2021). The GO30140 trial also evaluated atezolizumab in combination with bevacizumab, showing an objective remission rate (ORR) of 36% by independent review facility (IRF) based on RECIST 1.1 criteria and 39% by IRF using HCC mRECIST criteria, with a median OS of 17.1 months and no new safety concerns related to the combination therapy (Finn et al., 2020; Cheng et al., 2022; Lee et al., 2020).

The ORIENT-32 study demonstrated superior OS and PFS with sindilizumab plus bevacizumab over sorafenib alone, with respective medians not reached and 10.4 months for OS (HR = 0.569, 95% CI 0.431–0.751, p<0.0001) and 4.5 vs 2.8 months for PFS (HR = 0.567, 95% CI 0.457–0.704, p<0.0001) (Ren et al., 2021). Conversely, the CheckMate 459 study did not show significant prolongation of OS but reported a higher ORR (Yau et al., 2022). Notably, previous HCC studies have highlighted better outcomes with immunotherapy in patients with hepatitis virus infection; the CheckMate 459 cohort, which included more patients without hepatitis virus infection, exhibited diminished efficacy with nivolumab, potentially affecting overall efficacy (Yau et al., 2022).

The KEYNOTE-524 study investigated lenvatinib with pembrolizumab, revealing an ORR of 46% by mRECIST and 36% by RECIST v1.1 criteria, with 83% of patients experiencing target lesion reduction. However, there was a high incidence of grade 3 or higher treatment-related adverse events at 67%, including 3% of drug-related deaths (Sun et al., 2022).

#### Second-Line Setting

The RATIONALE 208 study, an open-label, global, multicenter phase II clinical trial, observed that patients with advanced HCC previously treated with sorafenib/lenvatinib systemic therapy exhibited an independently reviewed ORR of 13.6% (95% CI: 9.5, 18.7) and a median OS of 13.5 months (95% CI: 10.9, 15.8) after a median follow-up of 12.5 months. Tirilizumab was well tolerated, with generally low severity of adverse events (Table 1; Serrano et al., 2022; Ren et al., 2023).

Similarly, pembrolizumab demonstrated an ORR of 18.3% and an OS of 13.9 months in the global Phase III KEYNOTE-240 trial (Table 1). These findings are consistent with earlier phase II studies involving nivolumab in CheckMate 040 and pembrolizumab in KEYNOTE-224 as second-line treatments for advanced HCC, showing ORRs ranging from 13.8% to 15.7% and median OS durations of 12.9–15 months (Table 1; Sangro et al., 2020; Yau et al., 2020; Kudo et al., 2021). RESCUE is a domestic multicenter phase II clinical study in which all patients received karelizumab in combination with apatinib. The results also showed better anti-tumor activity and safety (Table 1; Xu et al., 2021).

These clinical trials underscore the unique immunoregulatory role of immune checkpoints within the tumor immune microenvironment. They target various markers, application phases, and sequences, thereby benefiting distinct clinical populations. The limited responsiveness to immunotherapy further highlights the necessity for comprehensive analysis of the HCC immune microenvironment to modulate immune resistance mechanisms, which could inform the development of novel therapeutics and facilitate translational advancement.

### Conclusion and Future Perspective

In summary, the immune microenvironment of HCC is composed of immune cells, fibroblasts, and stromal cells, which play an important role in tumor cell proliferation, invasion, and angiogenesis (Hanahan and Weinberg, 2011). The phenomenon of resistance to immunotherapy in HCC is primarily due to the intricate regulatory network within the TME. The results of single-cell sequencing analysis reflect the spatiotemporal specificity of TME and the dynamic change of cellular composition ratio. Moreover, the data suggest that because of tumor heterogeneity, targeting the TME may be a more effective strategy than targeting tumor cells (Lujambio et al., 2013; Zhu et al., 2022). HCC patients can benefit from various therapeutic approaches that consider the immune characteristics of the TME. Several avenues of research and development can be considered.

#### Biomarker Identification

Investigate novel biomarkers associated with HCC immune response and immunotherapy outcomes. This could include identifying immune cell signatures, TME characteristics, or genetic markers that predict response to immunotherapy.

#### Personalized Medicine

Develop personalized treatment strategies based on the individual patient’s tumor characteristics, immune profile, and genetic makeup. This may involve the use of biomarkers to tailor treatment selection and optimize patient outcomes.

#### Immune Checkpoint Inhibitors

Continue to evaluate the efficacy and safety of ICIs (e.g. anti-PD-1, anti-PD-L1, anti-CTLA-4) in HCC, both as monotherapy and in combination with other agents. Investigate novel immune checkpoints and immune checkpoint combinations for improved responses. Furthermore, in conjunction with AlphaFold3 and its associated applications, computer-aided target prediction can assist in narrowing down the range of potential targets for identification (Abramson et al., 2024).

#### Preclinical Models and Translational Research

Utilize advanced preclinical models, patient-derived organoids, and patient-derived xenografts to better understand the complex interactions between the immune system and HCC tumors. Translate preclinical findings into clinical trials and patient care.

#### Clinical Trials and Data Analysis

Conduct well-designed clinical trials to evaluate the safety and efficacy of novel immunotherapy approaches in HCC patients. Analyze clinical trial data to identify predictors of response, mechanisms of resistance, and potential biomarkers for patient stratification.

By pursuing these research directions and leveraging advances in immunotherapy, precision medicine, and translational oncology, we can enhance our understanding of HCC immunotherapy and improve treatment outcomes for patients with this challenging disease.
